# Integrated analysis of microbiota and gut microbial metabolites in blood for breast cancer

**DOI:** 10.1128/msystems.00643-24

**Published:** 2024-10-18

**Authors:** Yu Peng, Jiale Gu, Fubin Liu, Peng Wang, Xixuan Wang, Changyu Si, Jianxiao Gong, Huijun Zhou, Ailing Qin, Fangfang Song

**Affiliations:** 1 Department of Epidemiology and Biostatistics, Key Laboratory of Molecular Cancer Epidemiology, Key Laboratory of Prevention and Control of Major Diseases in the Population, Ministry of Education, National Clinical Research Center for Cancer, Tianjin Medical University Cancer Institute and Hospital, Tianjin Medical University, Tianjin, China; APC Microbiome Ireland, Cork, Ireland

**Keywords:** blood microbiota, gut microbial metabolites, breast cancer, composite diagnostic panel

## Abstract

Gut microbiota and associated metabolites have been linked to breast carcinogenesis. Evidences demonstrate blood microbiota primarily originates from the gut and may act as a biomarker for breast cancer. We aimed to characterize the microbiota–gut microbial metabolites cross-talk in blood and develop a composite diagnostic panel for breast cancer. We performed 16S rRNA gene sequencing and metabolomics profiling on blood samples from 107 breast cancer cases and 107 age-paired controls. We found that the alpha diversity of the blood microbiota was decreased in breast cancer compared to controls. There were significantly different profiles of microbiota and gut microbial metabolites in blood between these two groups, with nine bacterial genera and four gut microbial metabolites increased in patients, while thirty-nine bacterial genera and two gut microbial metabolites increased in controls. Some breast cancer-associated gut microbial metabolites were linked to differential blood microbiota, and a composite microbiota–metabolite diagnostic panel was further developed with an area under the curve of 0.963 for breast cancer. This study underscored the pivotal role of microbiota and gut microbial metabolites in blood and their interactions for breast carcinogenesis, as well as the potential of a composite diagnostic panel as a non-invasive biomarker for breast cancer.

**IMPORTANCE:**

Our integrated analysis demonstrated altered profiles of microbiota and gut microbial metabolites in blood for breast cancer patients. The extensive correlation between microbiota and gut microbial metabolites in blood assisted the understanding of the pathogenesis of breast cancer. The good performance of a composite microbiota–gut microbial metabolites panel in blood suggested a non-invasive approach for breast cancer detection and a novel strategy for better diagnosis and prevention of breast cancer in the future.

## INTRODUCTION

In 2020, there were an estimated 2.3 million breast cancer cases and 680,000 deaths in the world, and breast cancer incidence in China showed a rapidly increasing trend, resulting in a huge disease and economic burden (1). The traditional risk factors for breast cancer include age, lifestyle factors, reproductive and hormonal factors, and genetic factors (2). Alternatively, recent studies indicate that the tumor microbiota differs between healthy and breast cancer tissue, which could be involved in the carcinogenesis (3). Meanwhile, gut microbiota may translocate to breast tissue through dendritic cells and systemic circulation (4). The available data mostly inform that gut microbiota can drive breast cancer initiation and progression by influencing estrogen metabolism, immune system function, and gut microbial metabolites (5–7). Furthermore, microbiota located in the gut and breast tissue has the potential to be a diagnostic biomarker of breast cancer (3, 6, 8), which, however, is limited by the difficulty in obtaining these samples.

Generally, a blood sample is a commonly used and invasive sample and is considered a “sterile” environment in healthy individuals. However, with advances in molecular techniques and bioinformatics, blood microbiota has been uncovered in healthy individuals (9–11). A study based on healthy people does not support the existence of a core microbiota in blood but suggests the translocation of commensal microbiota into the blood from other body sites (12). Although the source of blood microbiota remains largely unknown, studies have hypothesized that it may originate from the gut (10, 11, 13). When the intestinal permeability increased, gut microbiota could be translocated to the circulatory system, and even if the intestinal epithelial membrane function was not compromised, dendritic cells, intestinal mucus-secreting goblet cells, and intestinal M-cells may facilitate the transfer of gut microbiota (10, 13). In addition, it remains to be further determined whether the microbiota in blood is alive and functional (14, 15). Currently, the blood microbiome has been studied in a variety of noncommunicable diseases, such as cardiovascular diseases, diabetes, and cancers (13, 16–23), while only two studies demonstrated that the blood microbiota altered in breast cancer (22, 23) and may act as a biomarker for early diagnosis of breast cancer (23). Therefore, more research is needed to determine the importance of blood microbiota in the pathogenesis of breast cancer and their potential as early diagnostic biomarkers.

On the other hand, although the association between gut microbiota and breast cancer has been reported (5–8), the functional contribution of gut microbiota to breast cancer has not been characterized. As biochemical converters, microbial metabolites connect gut microbiota to breast cancer (5, 24). Short-chain fatty acids (SCFAs) are produced through saccharolytic fermentation of carbohydrates by gut microbiota (25, 26), which can induce apoptosis through oxidative stress and may also reduce the invasive potential of breast cancer cells (27, 28). Lithocholic acid, a secondary bile acid produced by gut microbiota from primary bile acids, could inhibit the proliferation and invasion of breast cancer cells through TGR5 (29). However, little is known regarding the characteristics of gut microbial metabolites in blood and their associations with blood microbiota along breast carcinogenesis.

Therefore, we depicted the characteristics of microbiota and gut microbial metabolites in blood for breast cancer and explored microbiota–metabolite cross-talk in breast carcinogenesis. Meanwhile, we developed a composite diagnostic panel integrating microbiota and gut microbial metabolites in blood so as to provide new insights for the diagnosis of breast cancer in the future.

## MATERIALS AND METHODS

### Study population and sample collection

The present study included 107 breast cancer cases from the Tianjin Medical University Cancer Institute and Hospital and 107 age-paired healthy controls from the Chinese Colorectal, Breast, Lung, Liver, And Stomach cancers Screening Trial (C-BLAST) (30). The inclusion criteria of cases were as follows: (i) Tianjin residents, aged 18–65 years old, with newly diagnosed and histologically confirmed breast cancer since 1 January 2003; (ii) without receiving any treatments before admission; (iii) no history of any malignant tumors; (iv) no history of blood transfusion 6 months before blood collection. This study protocol was approved by the Ethics Committee of Tianjin Medical University Cancer Institute and Hospital, and all participants provided written informed consent. Baseline information and clinicopathological data were collected through structured questionnaires and medical records. Fasting blood samples were collected for evaluation of breast cancer cases and controls on the day of admission and screening, respectively. Blood samples were centrifuged to obtain plasma samples and stored at −80℃ for standby.

### Blood microbiota sequencing

DNA from the plasma samples was extracted using the cetyltrimethyl ammonium bromide (CTAB) method according to the manufacturer’s instructions. The total DNA was eluted in 50 µL of elution buffer and stored at −80°C until measurement in the PCR by LC-Bio Technology Co., Ltd, Hangzhou, Zhejiang Province, China. Variable regions V3–V4 of the bacterial 16S rRNA gene were amplified with bacterial PCR primers, 341F (5′-CCTACGGGNGGCWGCAG-3′) and 805R (5′-GACTACHVGGGTATCTAATCC-3′). PCR amplification was performed in a total volume of 25 µL of the reaction mixture containing 25 ng of template DNA, 12.5 µL PCR Premix, 2.5 µL of each primer, and PCR-grade water to adjust the reaction volume. The PCR products were confirmed with 2% agarose gel electrophoresis. Throughout the DNA extraction process, ultrapure water, instead of a sample solution, was used to exclude the possibility of false-positive PCR results. Subsequently, the PCR products were purified by AMPure XT beads (Beckman Coulter Genomics, Danvers, MA, USA) and quantified by Qubit (Invitrogen, USA). The size and quantity of the amplicon library constructed from purified PCR products were assessed on an Agilent 2100 Bioanalyzer (Agilent, USA) and with the Library Quantification Kit for Illumina (Kapa Biosciences, Woburn, MA, USA), respectively. Finally, the libraries were sequenced on the NovaSeq PE250 platform. At the same time, sterile DNA-free water was used as a negative control in PCR amplification and sequencing library preparation for subsequent identification of reagent contaminants during the experiment.

### Microbiota data analysis

Paired-end reads were truncated by cutting off the barcode and primer sequence and merged using FLASH (v1.2.8). Quality filtering of the raw reads was performed according to the fqtrim (v0.94), and chimeric sequences were filtered out using Vsearch (v2.3.4). The QIIME2 was used for dereplication to obtain amplicon sequence variations (ASVs). We identified and removed potential contaminants using the decontam package (v.1.2.1) by the prevalence of each ASV in blood samples compared to the prevalence in negative controls, and this method is suitable for low-biomass samples. Then, we calculated the alpha diversity (Shannon and Simpson index) and beta diversity (weighted UniFrac distance). Differences in the beta diversity between groups were examined and illustrated by permutational multivariate analysis of variance (PERMANOVA) and a principal coordinates analysis (PCoA) diagram. Then, ASVs were annotated with the SILVA database (release 138) for each representative sequence. Linear discriminant analysis effect size (LEfSe) was used to identify differential blood microbiota between cases and controls. Based on the Kyoto Encyclopedia of Genes and Genomes (KEGG), we further predict the functional profile of microbial communities by PICRUSt2.

### Metabolomics profiling

Untargeted metabolomic profiling was used to detect levels of metabolites in the plasma. A mixture (450 µL) of methanol–acetonitrile (acetonitrile: water = 9:1, 2% methanol) was added to the plasma sample (150 µL) and vortexed for 15 minutes. The supernatant of each sample was collected after the mixture was allowed to stand for 10 minutes at 4°C and centrifuged at 13,000 rpm for 10 miutes. The metabolite profiling for plasma was performed on the Nexera X2 system (SHIMADZU, Japan) coupled with Triple TOF 5600+ (AB SCIEX, USA). Briefly, derivatized samples were eluted through an Agilent ZORBAX Eclipse Plus C18 (2.1 × 100 mm, 3.5 um) at a temperature of 35°C and a flow rate of 0.5 mL/min (mobile phase A was 0.1% formic acid in water, and mobile phase B was acetonitrile). The mass spectrometer (MS) was equipped with an electrospray ionization source and operated in both positive and negative ion modes. The parameters of MS were as follows: source temperature, 120°C; desolvation temperature, 500°C; desolvation gas flow, 600 L/h; cone gas flow, 50 L/h; capillary voltage, 3.0 kV for the positive ion mode and 4.5 kV for negative ion mode; sampling cone voltage, 27 eV; extraction cone voltage, 4 eV; scanning range, m/z 50–1,500.

### Metabolomics data analysis

The raw data were imported to MarkerView software to preprocess, such as retention time alignment, peak discrimination, filtering, and alignment, and generate a peak table with retention time (*t*_*R*_), *m*/*z* value, and corresponding peak intensity. Using the Human Metabolome Database (HMDB), we identified metabolites related to gut microbiota in blood. After excluding outliers (mean ± 4 standard deviations [SD]), data of gut microbial metabolites were standardized. Differential gut microbial metabolite analyses were conducted using partial least square discriminant analysis (PLS-DA) and Student’s t test, followed by an enrichment analysis based on the KEGG database using the MetaboAnalyst 6.0 program (http://www.metaboanalyst.ca).

### Statistical analysis

Baseline characteristics of this study population were summarized using the mean ± SD for quantitative variables and number (percentage) for categorical variables and examined by Student’s *t* test and χ^2^ test, respectively. We observed the correlation between the differential microbiota and gut microbial metabolites in blood by Spearman’s correlation. The tenfold cross-validation was conducted to identify optimal microbiota and related gut metabolite biomarkers in blood using the random forest model, and receiver operating characteristic (ROC) curve and area under curve (AUC) were used to evaluate the performance of biomarkers. The false discovery rate (FDR) was used for multiple comparisons. SAS (version 9.4, SAS Institute Inc, Cary, NC) and R software (version 4.2.3) were used for statistical analysis. A two-sided *P* value < 0.05 was considered statistically significant.

## RESULTS

### Characteristics of the study population

This study initially included 107 breast cancer cases and 107 healthy controls for microbiome and metabolome sequencing. After quality control, studies of blood microbiota and microbial metabolites included 195 (88 cases and 107 controls) and 101 (98 cases and 93 controls) participants, respectively (Fig. S1). In the total population, compared with the healthy controls, individuals with breast cancer had a higher prevalence of obesity and a higher proportion of experiencing negative events, history of abortion and benign breast diseases, and had lower income (*P* < 0.05, Table 1). Generally, the baseline characteristics of individuals in studies of blood microbiota and microbial metabolites were similar to those in the total population (*P* < 0.05, Table S1).

**TABLE 1:** Baseline characteristics of the study population^*a*^

Characteristics	Control (*N* = 107）	Case (*N* = 107）	*P* value
Age (mean ± SD, year)	53.3 ± 5.8	53.3 ± 6.0	0.925
BMI, *n* (%)			<0.001
≤23.9	55 (51.89)	35 (32.71)	
24–27.9	43 (40.57)	46 (42.99)	
≥28	8 (7.55)	26 (24.30)	
Education, *n* (%)			0.043
Primary school or below	5 (4.67)	15 (15.00)	
Junior or senior high school	77 (71.96)	64 (64.00)	
Junior college or above	25 (23.36)	21 (21.00)	
Income, *n* (%)			0.001
<1,000	8 (7.55)	19 (19.59)	
1,000–2,999	65 (61.32)	67 (69.07)	
≥3,000	33 (31.13)	11 (25.00)	
Smoking, *n* (%)			0.672
No	97 (96.04)	101 (97.12)	
Yes	4 (3.96)	3 (2.88)	
Drinking, *n* (%)			0.984
No	100 (98.04)	102 (98.08)	
Yes	2 (1.96)	2 (1.92)	
Negative events, *n* (%)			0.005
No	89 (89.90)	73 (74.49)	
Yes	10 (10.10)	25 (25.51)	
Age of menarche, *n* (%)			0.114
≤13	18 (17.14)	27 (25.23)	
14	16 (15.24)	25 (23.36)	
15	23 (21.90)	16 (14.95)	
≥16	48 (45.71)	39 (36.45)	
Abortion, *n* (%)			<0.001
No	42 (41.18)	18 (17.31)	
Yes	60 (58.82)	86 (82.69)	
Menopause, *n* (%)			0.995
No	33 (32.04)	34 (32.08)	
Yes	70 (67.96)	72 (67.92)	
HRT use, *n* (%)			0.232
No	85 (94.44)	87 (89.69)	
Yes	5 (5.56)	10 (10.31)	
Oral contraceptive use, *n* (%)			0.633
No	84 (82.35)	84 (84.85)	
Yes	18 (17.65)	15 (15.15)	
History of benign breast disease, *n* (%)			0.006
No	83 (82.18)	66 (65.35)	
Yes	18 (17.82)	35 (34.65)	
Family history of breast cancer, *n* (%)			0.053
No	104 (99.05)	98 (94.23)	
Yes	1 (0.95)	6 (5.77)	

^*a*^ Abbreviations: BMI, body mass index; HRT, hormone replacement therapy; SD, standard deviation.

### Characterization of blood microbiota in breast cancer

**Fig 1:**
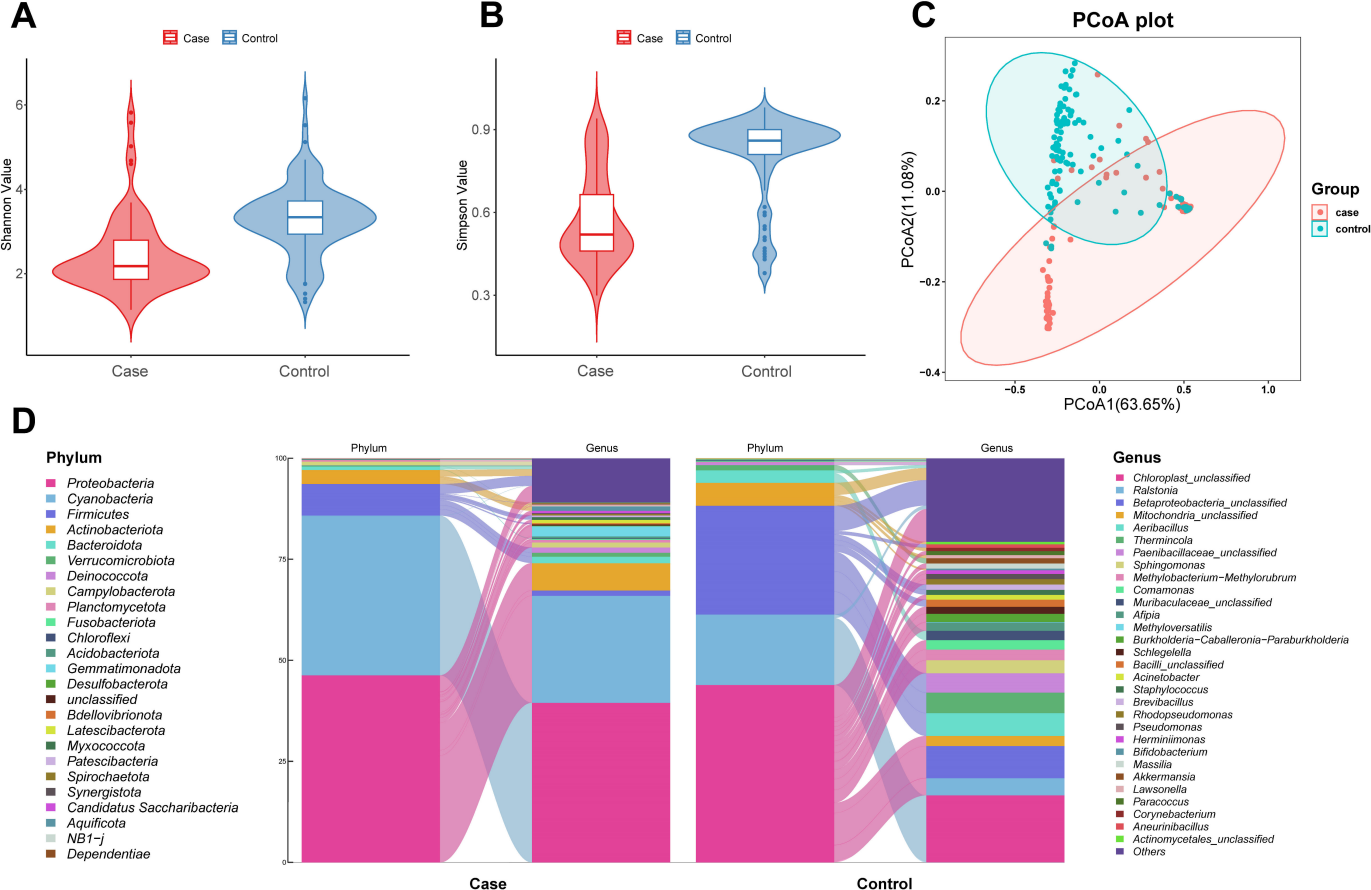
Diversity and composition of the blood microbiota in breast cancer cases and controls. (**A**) Alpha diversity estimated by Shannon index and (**B**) Simpson index. (**C**) Beta diversity calculated by PCoA of weighted UniFrac distances. (**D**) Microbial composition from the phylum level to genus level in breast cancer cases and controls. Abbreviation: PCoA, principal coordinate analysis.

At the ASV level, there were 613 shared ASVs in breast cancer cases and controls, 2,097 case-specific ASVs, and 1,179 control-specific ASVs (Fig. S2). The number of ASVs in controls was lower than that in the breast cancer cases. The results of Shannon and Simpson index indicated that alpha diversity was decreased in breast cancer patients than in healthy controls (*P* < 0.05, Fig. 1A and B). In terms of beta diversity, the PCoA and PERMANOVA test revealed a significant difference in the overall blood microbial composition between the two groups (*P* < 0.001, Fig. 1C). At the phylum level, we observed a domination by members of *Proteobacteria* and *Firmicutes* in these two groups (Fig. 1D). At the genus level, compared to healthy controls, *Ralstonia* within phylum *Proteobacteria* was more abundant in cases, but *Aeribacillus* and *Thermincola* within phylum *Firmicutes* was less abundant. Simultaneously, we found that the known genus with the highest relative abundance was *Ralstonia,* followed by *Methyloversatilis* and *Bifidobacterium* in breast cancer patients, and *Aeribacillus* was followed by *Thermincola* and *Ralstonia* in the healthy controls (Fig. 1D; Fig. S3). These results suggested that the breast cancer patients had a unique blood microbiota composition compared with the healthy controls.

The functional features of the blood microbiota in the two groups were further investigated according to the KEGG modules, and there were 28 KEGG modules with significant differences between patients and controls (FDR <  0.05, Fig. S4). Compared with the controls, the blood microbiota of cases were highly enriched in energy metabolism, replication and repair, as well as metabolism of cofactors and vitamins.

**Fig 2:**
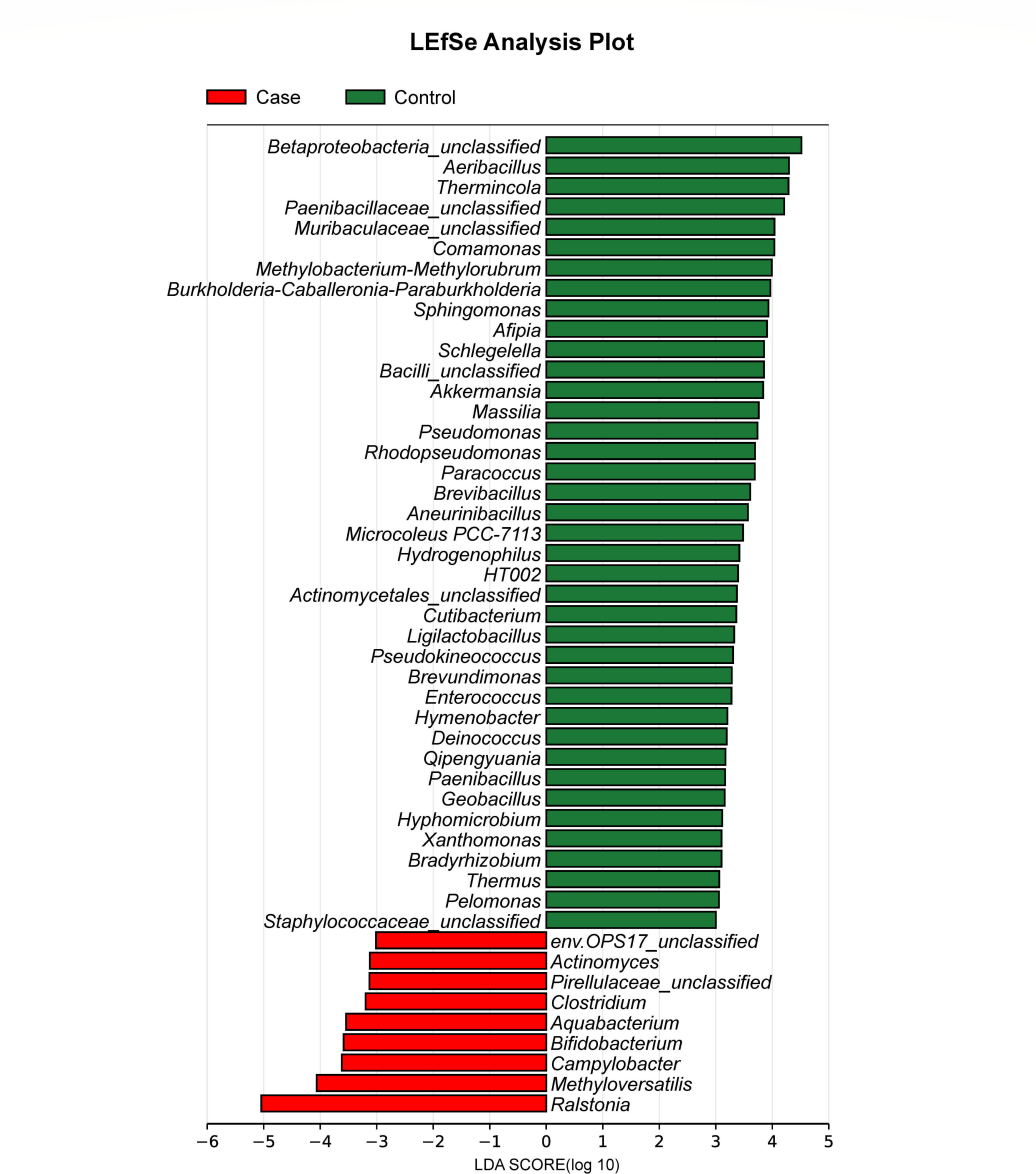
Differential blood microbiota between breast cancer cases and healthy controls. Abbreviation: LDA, linear discriminant analysis.

To further identify differential blood microbiota between breast cancer and controls, we focused on the genus levels to perform LEfSe analysis. Based on a linear discriminant analysis (LDA) score  >3.0, we observed 48 differential bacterial genera between the two groups, among which nine bacterial genera were significantly enriched in patients, including *Ralstonia*, *Methyloversatilis,* and *Campylobacter*, while 39 bacterial genera were significantly increased in healthy controls, such as *Aeribacillus*, *Thermincola,* and *Comamonas* (Fig. 2).

### Alterations of gut microbial metabolites in blood for breast cancer

**Fig 3:**
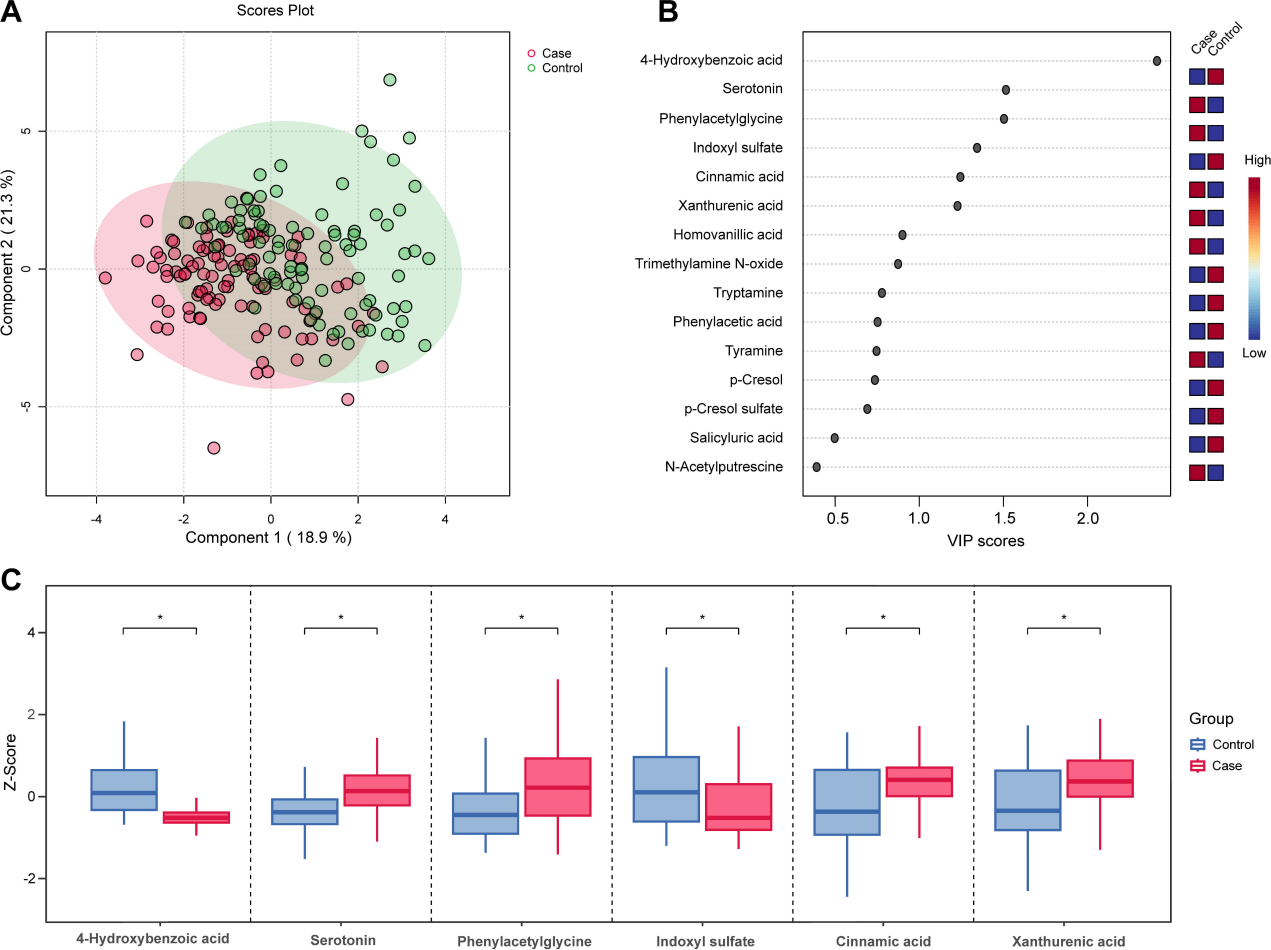
Distribution of gut microbial metabolites in blood between breast cancer cases and healthy controls. (**A**) Partial least squares-discriminant analysis (PLS-DA) for breast cancer cases and healthy controls. (**B**) The differences of gut microbial metabolites in blood displayed by the variable importance in projection (VIP) plot obtained from PLS-DA. (**C**) Levels of differential gut microbial metabolites in blood between breast cancer cases and healthy controls.

A total of 20 gut microbial metabolites in plasma were identified by HMDB, and there were differences in the expression of gut microbial metabolites between breast cancer cases and controls presented by hierarchical clustering (Fig. S5) and PLS-DA (Fig. 3A). According to a criterion of variable importance in projection (VIP) ≥1 and FDR < 0.05, six characteristic gut metabolites in blood with significantly different levels were identified (Fig. 3B; Table S1). With comparison to those in controls, the plasma levels of serotonin, phenylacetylglycine, cinnamic acid, and xanthurenic acid were higher, but the plasma levels of 4-hydroxybenzoic acid and indoxyl sulfate were lower in breast cancer groups (Fig. 3C). Furthermore, the functional analysis revealed that six differential gut metabolites in blood were distinctly enriched in the pathways of ubiquinone and biosynthesis of other terpenoid-quinone as well as tryptophan metabolism (Table S3, FDR < 0.05).

### Integrated analysis of microbiota and gut microbial metabolites in blood

**Fig 4:**
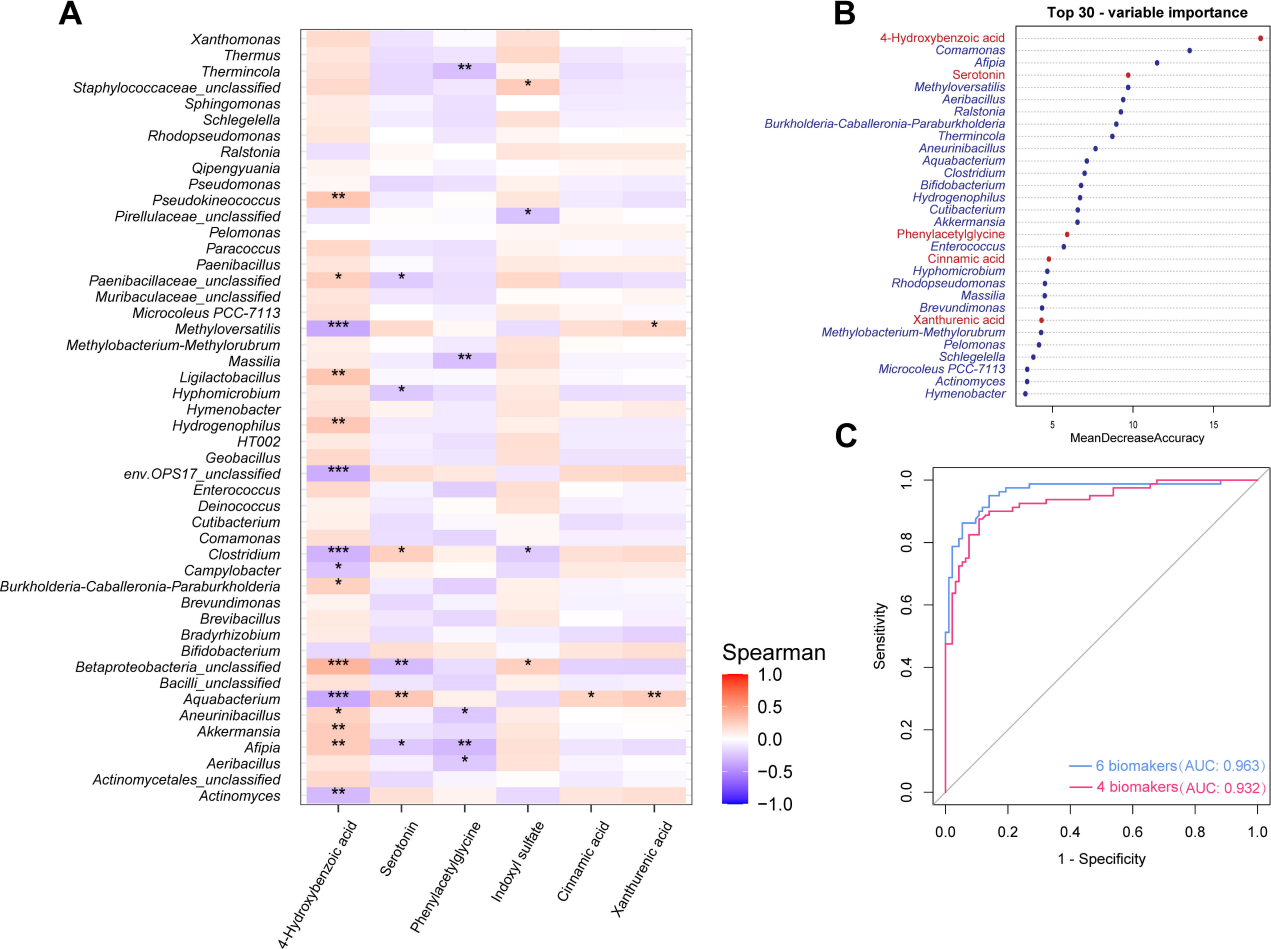
Integrated analysis of differential microbiota and gut microbial metabolites in blood. (**A**) The association between microbiota and gut microbial metabolites in blood. *: FDR < 0.05; **: FDR < 0.01; ***: FDR < 0.001. (**B**) Ranking of differential microbiota and gut microbial metabolites in blood according to the mean decreased accuracy. (**C**) Receiver operating characteristic curve (ROC) of the combined microbiota and gut microbial metabolite markers in blood. The panel for the red line included four microbiota (*Comamonas*, *Afipia*, *Methyloversatilis,* and *Aeribacillus*) and two gut microbial metabolites (4-hydroxybenzoic acid and serotonin) in blood, and the panel for the blue line included two blood microbiota (*Comamona* and *Aeribacillus*) and two microbial metabolites (4-hydroxybenzoic acid and serotonin).

The correlation analysis between the 48 differential genera and six altered gut microbial metabolites in blood suggested considerable microbiota–metabolite cross-talk involved in the carcinogenesis (Fig. 4A). In detail, decreased 4-hydroxybenzoic acid level in breast cancer patients was associated with low relative abundance of eight blood microbiota (for example, *Afipia*, *Ligilactobacillus*, *Hydrogenophilus,* and *Akkermansia*; β: 0.259–0.300; FDR < 0.05) and high relative abundance of six genera (such as *Methyloversatilis*, *Clostridium*, *Aquabacterium,* and *Actinomyces*; β: −0.370 to −0.300; FDR < 0.05). Meanwhile, there were positive associations of *Aquabacterium* with serotonin, cinnamic acid, and xanthurenic acid (β: 0.216–0.289, FDR < 0.05). *Clostridium* was positively correlated with serotonin (β: 0.251, FDR < 0.05), but negatively related to indoxyl sulfate (β:−0.222, FDR < 0.05).

We further performed a tenfold cross-validation on a random forest model to investigate the classification power for the combination of the microbiota and gut microbial metabolites in blood for breast cancer cases and controls. Considering the lower cross-validation error and the moderate number of markers, our analysis identified six optimal biomarkers including two gut microbial metabolites (4-hydroxybenzoic acid and serotonin) and four microbiotas in blood, namely, *Comamonas*, *Afipia*, *Methyloversatilis,* and *Aeribacillus* (Fig. S6; Fig. 4B), and the AUC value of this combined panel was 0.963 (Fig. 4C). Owing to the high correlation of 4-hydroxybenzoic acid with *Methyloversatilis* and *Afipia*, we further deleted *Methyloversatilis* and *Afipia* from the panel, with an AUC of 0.932.

## DISCUSSION

Our study indicated breast cancer patients had different profiles of microbiota and gut microbial metabolites in blood compared to healthy individuals, with significant alterations in 48 microbiota genera and six gut microbial metabolites. Integrated blood microbiome and metabolome analysis revealed extensive correlations between differential microbiota and gut microbial metabolites in blood. Furthermore, we constructed a composite diagnostic panel of blood microbiota–gut microbial metabolites that could be used for the diagnosis of breast cancer.

In recent years, blood microbiota has been reported to be associated with various diseases (9–12). To our knowledge, multiple studies have been conducted to investigate the role of blood microbiota on cancer risk to date, including colorectal (13, 20), gastric (21), lung (19), and breast cancers (22, 23). In this study, we observed a decreased alpha diversity of blood microbiota in breast cancer patients than that in healthy controls, which was also consistently observed in a Korean study (23), but contrary to the findings of a Chinese study (22). This may be due to the very small sample size of the Chinese study, with only five breast cancer cases included, and the fact that samples were plasma cell-free DNA. Simultaneously, the present study based on LEfSe analysis at the genus level also found that *Pseudomonas*, *Sphingomonas,* and *Cutibacterium* abundance decreased in healthy controls, whereas *Bifidobacterium* increased in breast cancer, as was reported by the Korean study (23). Additionally, we identified *Ralstonia*, *Methyloversatilis*, *Campylobacter*, *Aquabacterium*, *Clostridium*, and *Actinomyces* were enriched in breast cancer cases. Higher *Ralstonia* (31) and *Campylobacter* (32) abundance has been reported in breast cancer tissues compared to normal tissues, and another study revealed that the relative abundance of gut microbiota *Actinomyces* was increased in breast cancer cases (33). These consistent expressions of *Ralstonia*, *Campylobacter,* and *Actinomyce* in plasma and tissue/gut in breast cancer patients imply that these were key bacteria contributing to the pathogenesis of breast cancer, and there may be a gut–blood–tissue translocation route. *Clostridium* plays a crucial role in bile acid metabolism and could affect the occurrence and development of malignant tumors (34). An *in vitro* experiment showed that the *Clostridium*-specific metabolite, i.e., deoxycholic acid, could promote the growth of breast cancer cells (35). Therefore, these altered microbiota might have a crucial role in the initiation and development of breast cancer. Although a study supports the transfer of commensal microbiota from other body sites into the blood (12), the origin of the blood microbiota remains uncertain, which could be from the oral cavity, skin, or gastrointestinal tract (14, 36), and it remains unknown whether the blood microbiota is really functional (14, 15). Further studies are required to identify the origin of blood microbiota and their potential role in breast carcinogenesis.

In addition, many of the metabolites produced by the gut microbiota may be prominent factors involved in breast carcinogenesis, and there is probably extensive microbiota–metabolite cross-talk associated with the pathogenesis (37). By analyzing gut microbial metabolites in blood, we found that increased levels of serotonin, phenylacetylglycine, cinnamic acid, and xanthurenic acid, but decreased levels of 4-hydroxybenzoic acid and indoxyl sulfate in breast cancer cases compared with healthy controls. Different studies have demonstrated serotonin’s growth-stimulatory effect on breast cancer through the serotonin receptors (5-HT2A/C) (38, 39). A V-shaped dose–response curve of the 4-hydroxybenzoic acid concentration on breast cancer cell proliferation has been reported, which inhibited proliferation at specific concentrations (40). An *in vitro* study has shown that indoxyl sulfate, a tryptophan metabolite, acting through the aryl hydrocarbon receptor and pregnane-X-receptor, not only reduced the severity of breast cancer but also inhibited the proliferation of breast cancer cells (41). These studies further support our discovery in this study. Our population-based study supported that gut microbiota-associated metabolites may play important roles in breast carcinogenesis, but more efforts are needed to clarify the specific role of these metabolites in breast cancer. Meanwhile, the present study found some associations between a variety of differential gut microbial metabolites and microbiota in blood. Of note, there was a strong negative correlation of 4-hydroxybenzoic acid with *Methyloversatilis*, *Clostridium*, *Aquabacterium*, and *Actinomyces*. Serotonin was positively correlated with *Clostridium* and *Aquabacterium*. Collectively, these revealed the significant interactions between microbiota and gut microbial metabolites in blood on the occurrence of breast cancer.

Accumulating evidence has shed light on the possibility of screening potential biomarkers based on blood microbiota profiles to distinguish cancer patients and healthy controls (13, 19–21, 23). We further explored the potential value with the combination of microbiota and gut microbial metabolites in blood for the noninvasive diagnosis of breast cancer. We observed a higher discriminating power with an AUC of 0.963 by two microbial metabolites (4-hydroxybenzoic acid and serotonin) and four blood microbiota (*Comamonas*, *Afipia*, *Methyloversatilis,* and *Aeribacillus*). For the six biomarkers discovered in this study, the *Comamonas* in blood (21) and 4-hydroxybenzoic acid in urine (42) have been reported as possible biomarkers for gastric cancer, and another study indicated that plasma serotonin may serve as a biomarker for early detection of breast cancer recurrence (43), but the possibility of using other biomarkers for breast cancer detection has not been reported. Although more research investigations and clinical validations are needed, the composite microbiota–gut microbial metabolite panel in blood may help identify high-risk populations and also facilitate the translation of precision screening and clinical early diagnosis strategies.

This study based on the Chinese population was the first to depict the profiles of microbiota and gut microbial metabolites in blood for breast cancer and demonstrated a promising potential of the composite microbiota–gut microbial metabolite panel in blood for noninvasive diagnosis of breast cancer. However, the study has several limitations. First, although participants did not have symptoms of infection at the time of blood collection, healthy controls lacked a detailed medication history. Therefore, participants taking antibiotics or probiotics might not be excluded, thus affecting the results. Second, as this study was only a case–control study, residual confounding was possible, and it was unable to reveal the causal relationship. Third, although we took several measures to identify and control some potential contaminants during the experiment and analysis process, this still remains as a challenge faced by current technologies in the low-biomass blood samples. Finally, while the combination of microbiota and gut microbial metabolites in blood may serve as potential diagnostic biomarkers for breast cancer, further studies with larger sample sizes from multiple centers were needed to validate our findings.

In conclusion, our integrated analysis demonstrated the altered profiles of microbiota and gut microbial metabolites in blood for breast cancer patients. The extensive relationship between microbiota and gut microbial metabolites in blood assisted the understanding of the pathogenesis of breast cancer. The good performance of a composite microbiota–gut microbial metabolite panel in blood suggested a noninvasive approach for breast cancer detection and a novel strategy for better diagnosis and prevention of breast cancer in the future.

## Data Availability

The sequencing data supporting the conclusions of this article are available in the NCBI Sequence Read Archive (SRA) with the BioProject accession number PRJNA1162636.

## SUPPLEMENTAL MATERIAL

Supplemental material msystems.00643-24-s0001.doc Supplemental figures and tables.

ASM does not own the copyrights to Supplemental Material that may be linked to, or accessed through, an article. The authors have granted ASM a non-exclusive, world-wide license to publish the Supplemental Material files. Please contact the corresponding author directly for reuse.
