# Supplementary material for: Integrated analysis of microbiota and gut microbial metabolites in blood for breast cancer
Source: mSystems. 2024 Oct 18;9(11):e00643-24. doi: 10.1128/msystems.00643-24 (PMC11575300; doi:10.1128/msystems.00643-24)
Supplement: Supplemental material — Supplemental figures and tables. [file msystems.00643-24-s0001.doc]

**Integrated analysis of microbiota and gut microbial metabolites in blood for breast cancer**

**Supplementary Table:**

Supplementary Table 1. Baseline characteristics of the study population in the final analysis for microbiota and gut microbial metabolites in blood.

Supplementary Table 2. Differential gut microbial metabolites in blood between breast cancer cases and controls.

Supplementary Table 3. The functional analysis for six differential gut microbial metabolites in blood.

**Supplementary Figures:**

Supplementary Figure 1. Study design and flow diagram.

Supplementary Figure 2. The information of amplicon sequence variations between breast cancer cases and controls.

Supplementary Figure 3. The average relative abundance of the top 30 blood genera between breast cancer cases and controls.

Supplementary Figure 4. Functional alterations of blood microbiota between breast cancer cases and controls.

Supplementary Figure 5. Hierarchical clustering of gut microbial metabolites in blood between breast cancer cases and controls.

Supplementary Figure 6. A tenfold cross-validation on a random forest model to estimate the number of composite biomarkers predicting breast cancer risk.

**Supplementary Table 1. Baseline characteristics of the study population in the final analysis for microbiota and gut microbial metabolites in blood.**

| **Characteristics** | **Blood microbiota** | | | **Gut microbiota metabolites** | | |
| --- | --- | --- | --- | --- | --- | --- |
|  | **Control**  **(N=107)** | **Case**  **(N=88)** | ***P* value** | **Control**  **(N=93)** | **Case**  **(N=98)** | ***P* value** |
| **Age (mean±SD, year)** | 53.3±5.8 | 53.5±6.0 | 0.881 | 53.3±5.8 | 53.3±6.0 | 0.925 |
| **BMI, n (%)** |  |  | 0.002 |  |  | <0.001 |
| <=23.9 | 55 (51.89) | 28 (31.82) |  | 51 (54.84) | 32 (32.65) |  |
| 24-27.9 | 43 (40.57) | 40 (45.45) |  | 37 (39.78) | 41 (41.84) |  |
| >=28 | 8 (7.55) | 20 (22.73) |  | 5 (5.38) | 25 (25.51) |  |
| **Education, n (%)** |  |  | 0.155 |  |  | 0.017 |
| Primary school or below | 5 (4.67) | 10 (11.90) |  | 3 (3.23) | 14 (15.38) |  |
| Junior or Senior High School | 77 (71.96) | 53 (63.10) |  | 68 (73.12) | 57 (62.64) |  |
| Junior college or above | 25 (23.36) | 21 (25.00) |  | 22 (23.66) | 20 (21.98) |  |
| **Income, n (%)** |  |  | 0.008 |  |  | 0.001 |
| <1000 | 8 (7.55) | 13 (15.85) |  | 6 (6.52) | 17 (19.32) |  |
| 1000-2999 | 65 (61.32) | 58 (70.73) |  | 56 (60.87) | 61 (69.32) |  |
| >=3000 | 33 (31.13) | 11 (13.41) |  | 30 (32.61) | 10 (11.36) |  |
| **Smoking, n (%)** |  |  | 0.237 |  |  | 0.929 |
| No | 97 (96.04) | 85 (98.84) |  | 85 (97.70) | 93 (97.89) |  |
| Yes | 4 (3.96) | 1 (1.16) |  | 2 (2.30) | 2 (2.11) |  |
| **Drinking, n (%)** |  |  | 0.863 |  |  | 0.938 |
| No | 100 (98.04) | 84 (97.67) |  | 86 (97.73) | 93 (97.89) |  |
| Yes | 2 (1.96) | 2 (2.33) |  | 2 (2.27) | 2 (2.11) |  |
| **Negative events, n (%)** |  |  | 0.010 |  |  | 0.011 |
| No | 89 (89.90) | 62 (75.61) |  | 78 (90.70) | 68 (76.40) |  |
| Yes | 10 (10.10) | 20 (24.39) |  | 8 (9.30) | 21 (23.60) |  |
| **Age of menarche, n (%)** |  |  | 0.100 |  |  | 0.266 |
| <=13 | 18 (17.14) | 22 (25.00) |  | 16 (17.58) | 26 (26.53) |  |
| 14 | 16 (15.24) | 22 (25.00) |  | 14 (15.38) | 20 (20.41) |  |
| 15 | 23 (21.90) | 14 (15.91) |  | 20 (21.98) | 16 (16.33) |  |
| >=16 | 48 (45.71) | 30 (34.09) |  | 41 (45.05) | 36 (36.73) |  |
| **Breast feeding, n (%)** |  |  | 0.065 |  |  | 0.0154 |
| 0-12 | 57 (57.00) | 35 (43.21) |  | 49 (56.98) | 37 (38.64) |  |
| >12 | 43 (43.00) | 46 (56.79) |  | 34 (43.02) | 54 (61.36) |  |
| **Abortion, n (%)** |  |  | <0.001 |  |  | <0.001 |
| No | 42 (41.18) | 14 (16.28) |  | 37 (42.05) | 16 (16.84) |  |
| Yes | 60 (58.82) | 72 (83.72) |  | 51 (57.95) | 79 (83.16) |  |
| **Menopause, n (%)** |  |  | 0.840 |  |  | 0.917 |
| No | 33 (32.04) | 27 (30.68) |  | 30 (33.71) | 32 (32.99) |  |
| Yes | 70 (67.96) | 61 (69.32) |  | 59 (66.29) | 65 (67.01) |  |
| **HRT use, n (%)** |  |  | 0.186 |  |  | 0.232 |
| No | 85 (94.44) | 72 (88.89) |  | 72 (94.74) | 78 (88.64) |  |
| Yes | 5 (5.56) | 9 (11.11) |  | 4 (5.26) | 10 (11.36) |  |
| **Oral contraceptive use, n (%)** |  |  | 0.558 |  |  | 0.633 |
| No | 84 (82.35) | 71 (85.54) |  | 71 (80.68) | 76 (84.44) |  |
| Yes | 18 (17.65) | 12 (14.46) |  | 17 (19.32) | 14 (15.56) |  |
| **History of benign breast disease, n (%)** |  |  | 0.007 |  |  | 0.006 |
| No | 83 (82.18) | 55 (64.71) |  | 71 (81.61) | 62 (67.39) |  |
| Yes | 18 (17.82) | 30 (35.29) |  | 16 (18.39) | 30 (32.61) |  |
| **Family history of breast cancer, n (%)** |  |  | 0.118 |  |  | 0.062 |
| No | 104 (99.05) | 84 (95.45) |  | 90 (98.90) | 89 (93.68) |  |
| Yes | 1 (0.95) | 4 (4.55) |  | 1 (1.10) | 6 (6.32) |  |

Abbreviations: BMI, body mass index; HRT, hormone replacement therapy; SD, standard deviation.

**Supplementary Table 2. Differential gut microbial metabolites in blood between breast cancer cases and controls.**

| **Metabolites** | **t** | ***P* value** | **FDR** | **VIP**  **scores** |
| --- | --- | --- | --- | --- |
| **4-Hydroxybenzoic acid** | -7.833 | **<0.001^a^** | **<0.001^b^** | **2.413^c^** |
| **Serotonin** | 4.496 | **<0.001^a^** | **<0.001^b^** | **1.515^c^** |
| **Phenylacetylglycine** | 4.458 | **<0.001^a^** | **<0.001^b^** | **1.504^c^** |
| **Indoxyl sulfate** | -3.942 | **<0.001^a^** | **<0.001^b^** | **1.344^c^** |
| **Cinnamic acid** | 3.629 | **<0.001^a^** | **0.001^b^** | **1.244^c^** |
| **Xanthurenic acid** | 3.576 | **<0.001^a^** | **0.001^b^** | **1.227^c^** |
| **Homovanillic acid** | 2.583 | **0.011^a^** | **0.030^b^** | 0.900 |
| **Trimethylamine N-oxide** | -2.505 | **0.013^a^** | **0.033^b^** | 0.874 |
| **Tryptamine** | -2.222 | **0.027^a^** | 0.061 | 0.778 |
| **Phenylacetic acid** | -2.147 | **0.033^a^** | 0.062 | 0.752 |
| **Tyramine** | 2.128 | **0.035^a^** | 0.062 | 0.746 |
| **p-Cresol** | -2.100 | **0.037^a^** | 0.062 | 0.736 |
| **p-Cresol sulfate** | -1.969 | **0.050^a^** | 0.078 | 0.691 |
| **Salicyluric acid** | -1.409 | 0.160 | 0.229 | 0.497 |
| **N-Acetylputrescine** | 1.103 | 0.271 | 0.362 | 0.390 |
| **Equol** | -0.348 | 0.729 | 0.863 | 0.123 |
| **5-Hydroxyindoleacetic acid** | -0.336 | 0.737 | 0.863 | 0.119 |
| **Homovanillic acid sulfate** | -0.285 | 0.776 | 0.863 | 0.101 |
| **Indole** | 0.178 | 0.859 | 0.904 | 0.063 |
| **Indole-3-propionic acid** | 0.024 | 0.981 | 0.981 | 0.008 |

^a^：*P* value <0.05. ^b^：FDR <0.05. ^c^：VIP scores >1.

Abbreviations: FDR, false discovery rate; VIP, variable importance in projection.

**Supplementary Table 3. The functional analysis for six differential gut microbial metabolites in blood.**

| **Metabolite Set** | ***P* value** | **FDR** |
| --- | --- | --- |
| **Ubiquinone and other terpenoid-quinone biosynthesis** | 3.34E-13 | 6.68E-13 |
| **Tryptophan metabolism** | 1.21E-05 | 1.21E-05 |

Abbreviation: FDR, false discovery rate.

**
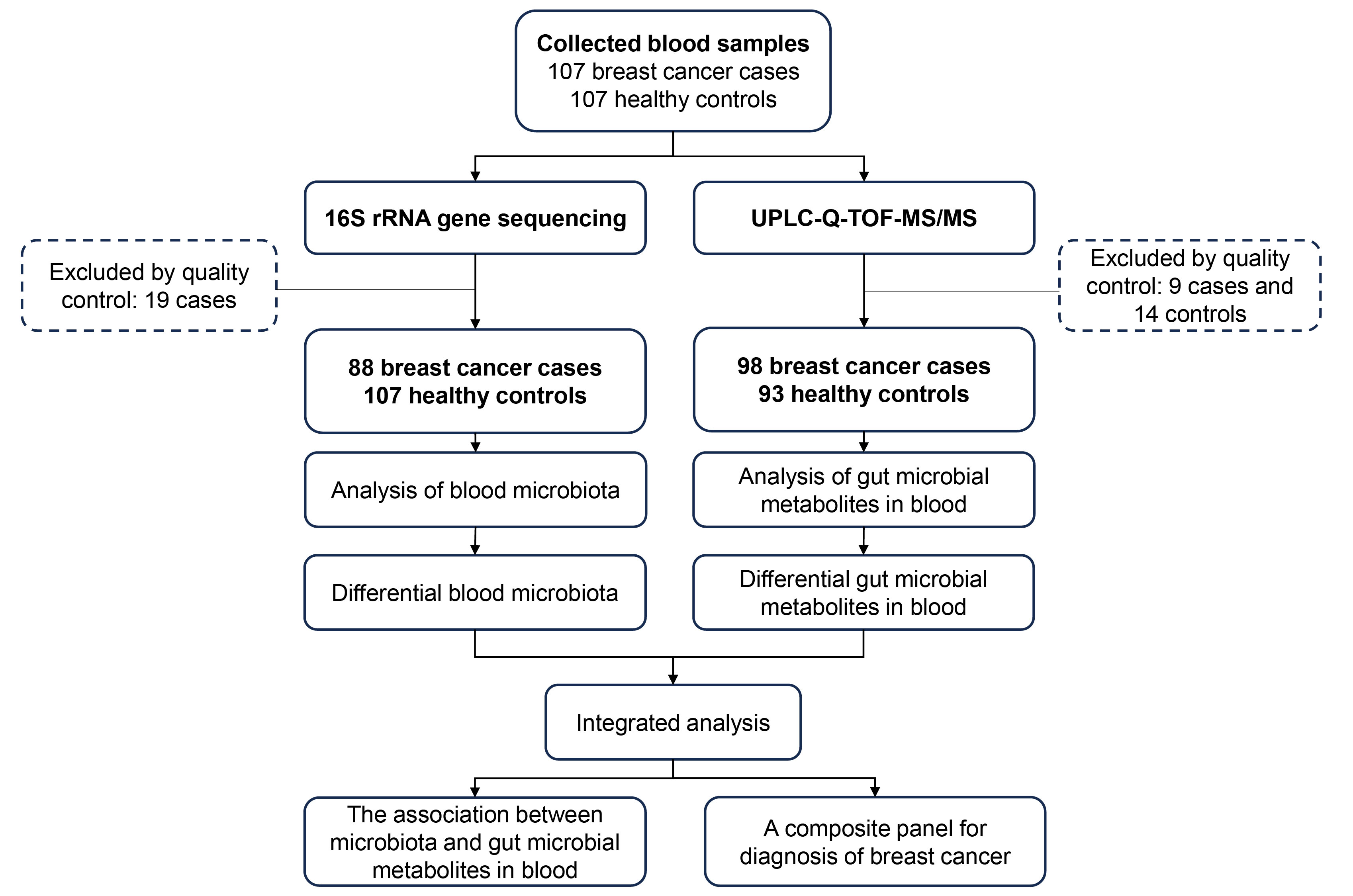
**

**Supplementary Figure 1. Study design and flow diagram.**


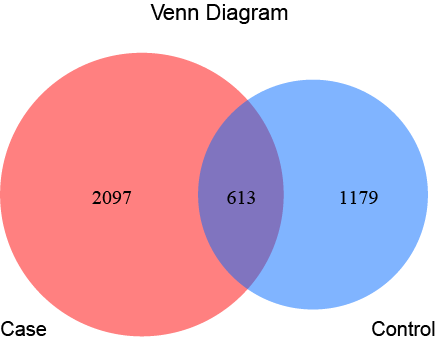


**Supplementary Figure 2. The information of amplicon sequence variations between breast cancer cases and controls.**


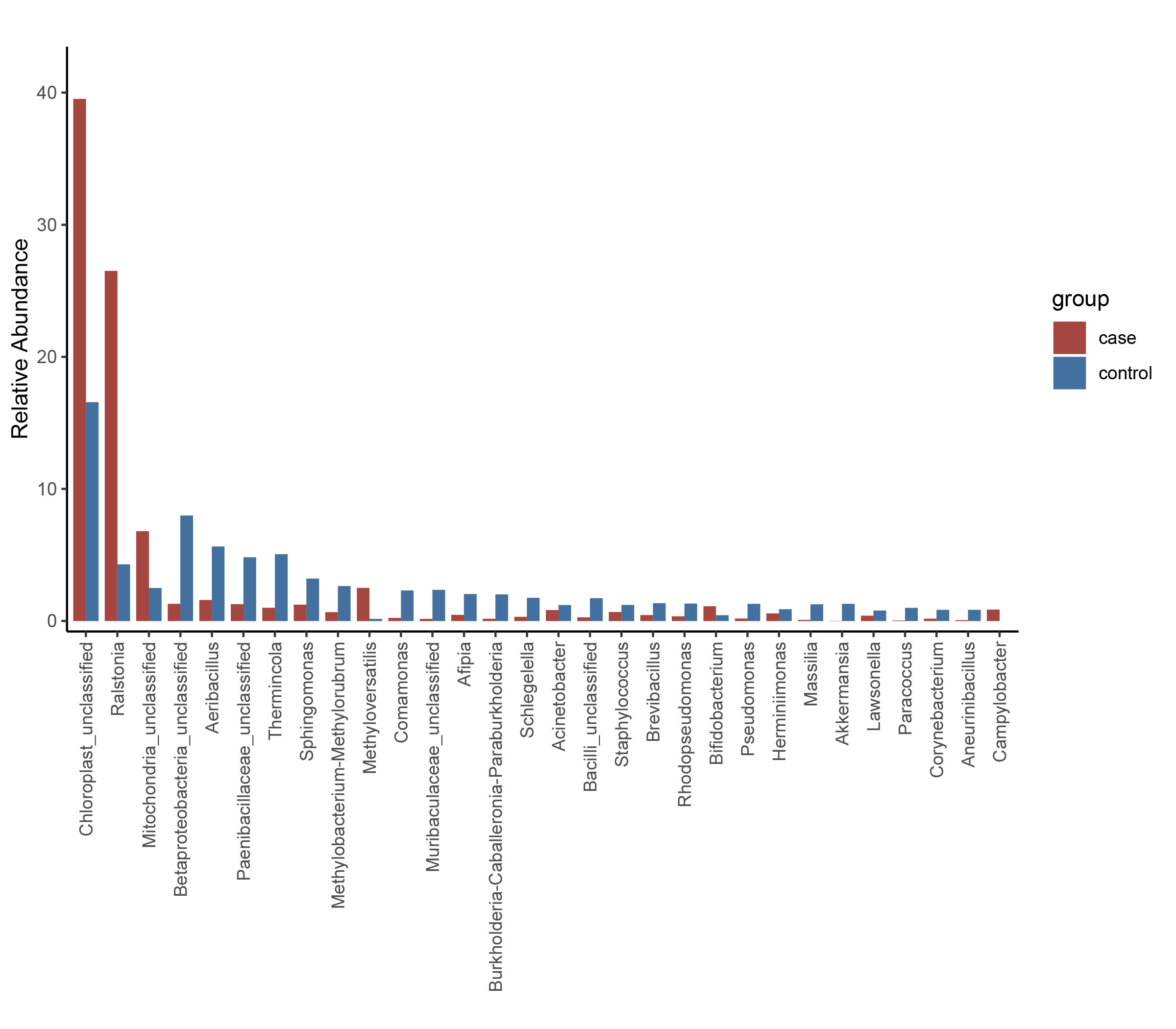


**Supplementary Figure 3. The average relative abundance of the top 30 blood genera between breast cancer cases and controls.**


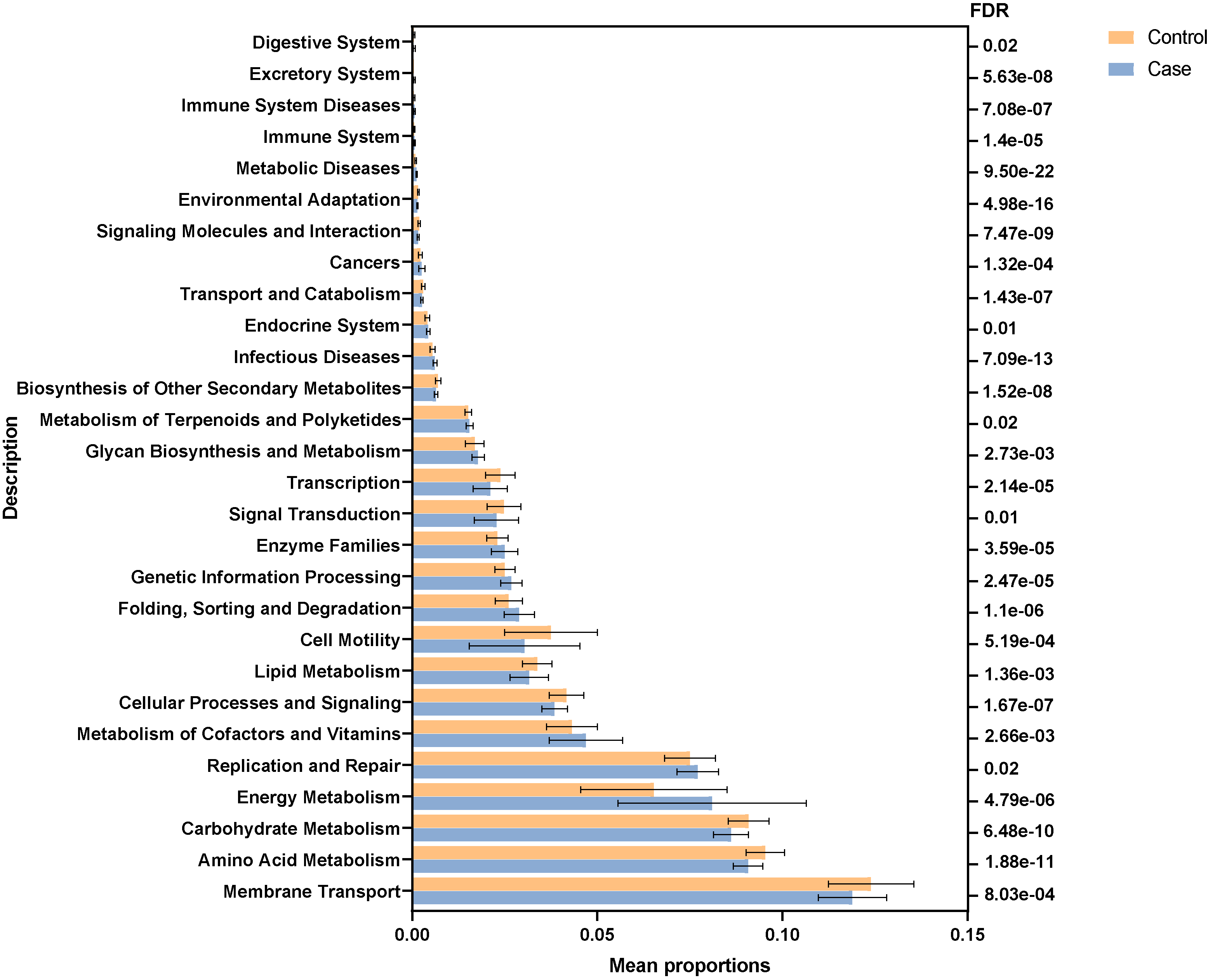


**Supplementary Figure 4. Functional alterations of blood microbiota between breast cancer cases and controls.**


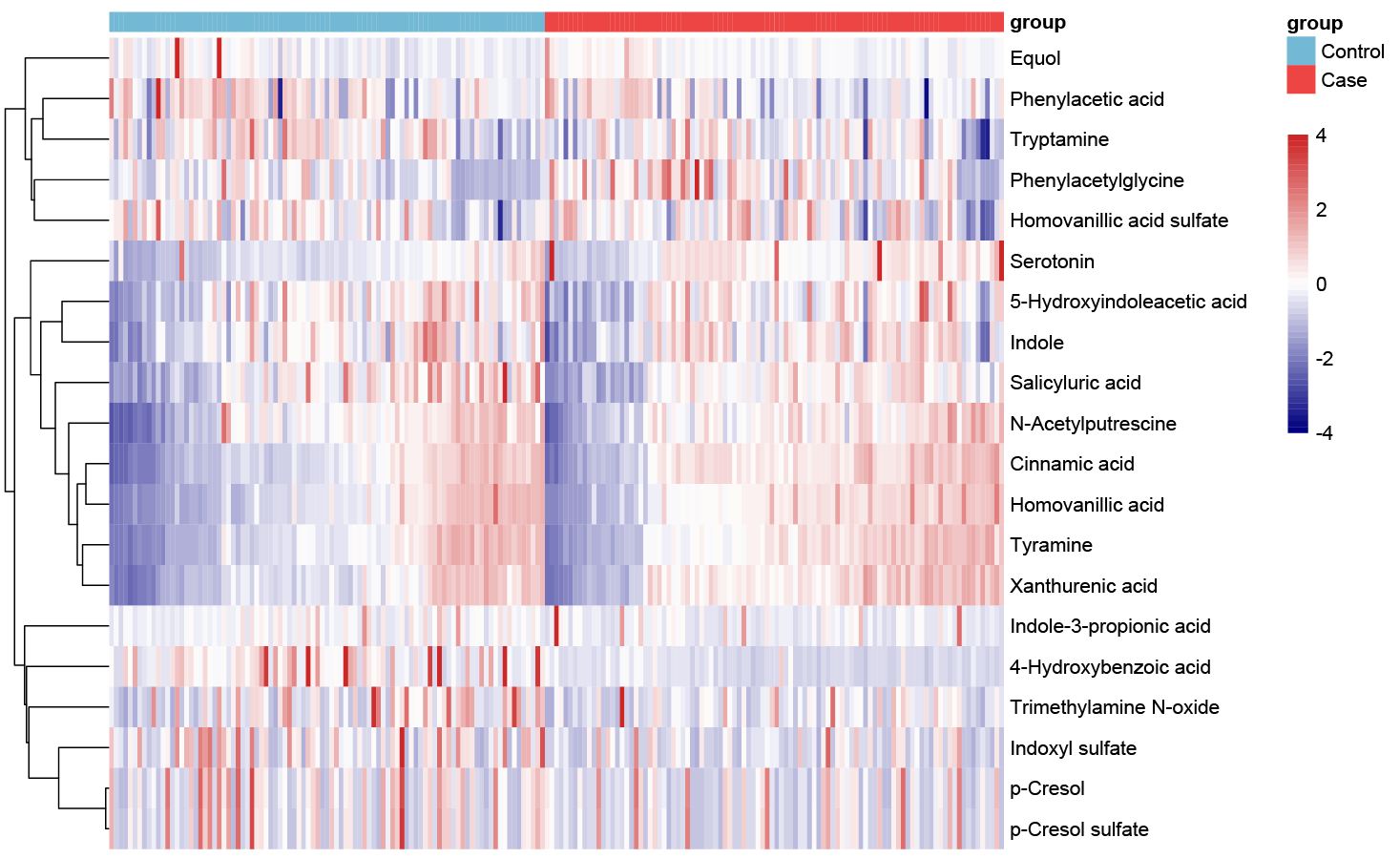


**Supplementary Figure 5. Hierarchical clustering of gut microbial metabolites in blood between breast cancer cases and controls.**


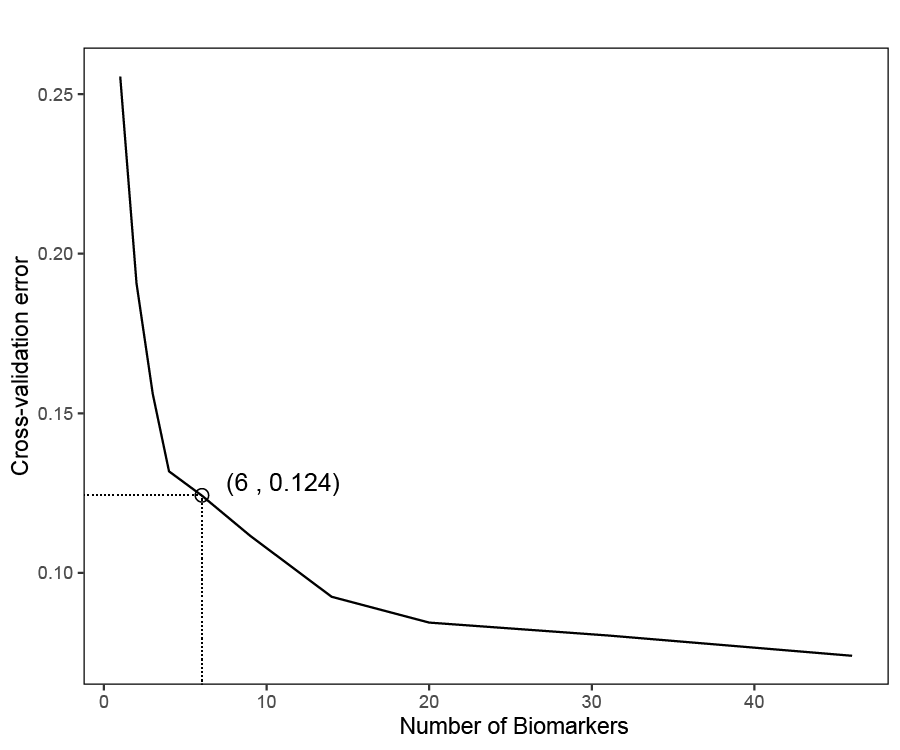


**Supplementary Figure 6.** **A tenfold cross-validation on a random forest model to estimate the number of composite biomarkers predicting breast cancer risk.**
